# The protective roles of augmenter of liver regeneration in hepatocytes in the non-alcoholic fatty liver disease

**DOI:** 10.3389/fphar.2022.928606

**Published:** 2022-10-11

**Authors:** Yuan Dong, Yuejie Zhang, Yingmei Feng, Wei An

**Affiliations:** ^1^ Department of Science and Technology, Beijing Youan Hospital, Capital Medical University, Beijing, China; ^2^ Department of Cell Biology, Capital Medical University and the Municipal Key Laboratory for Liver Protection and Regulation of Regeneration, Beijing, China

**Keywords:** augmenter of liver regeneration, non-alcohol fatty liver disease, non-alcoholic steatohepatitis, mitochondrion (mitochondria), hepatic/liver cells

## Abstract

Non-alcoholic fatty liver disease (NAFLD) occurs in 25% of the global population and manifests as lipid deposition, hepatocyte injury, activation of Kupffer and stellate cells, and steatohepatitis. Predominantly expressed in hepatocytes, the augmenter of liver regeneration (ALR) is a key factor in liver regulation that can alleviate fatty liver disease and protect the liver from abnormal liver lipid metabolism. ALR has three isoforms (15-, 21-, and 23-kDa), amongst which 23-kDa ALR is the most extensively studied. The 23-kDa ALR isoform is a sulfhydryl oxidase that resides primarily in the mitochondrial intermembrane space (IMS), whereby it protects the liver against various types of injury. In this review, we describe the role of ALR in regulating hepatocytes in the context of NAFLD. We also discuss questions about ALR that remain to be explored in the future. In conclusion, ALR appears to be a promising therapeutic target for treating NAFLD.

## Introduction

Non-alcoholic fatty liver disease (NAFLD) is a spectrum of diseases that initially manifest as non-alcoholic fatty liver (NAFL) or non-alcoholic steatohepatitis (NASH). NAFL is characterized as simple steatosis without histological evidence of hepatocyte injury or inflammation, whereas NASH occurs with the presence of hepatic inflammation and ballooning degeneration (Schwimmer et al., 2005). When further aggravated, NASH may progress toward liver fibrosis and subsequently drive progression to advanced stages, including cirrhosis, hepatic decompensation, and hepatocellular carcinoma (Kim et al., 2018; Huang et al., 2021a). The liver has a powerful regenerative ability in many vertebrates. The augmenter of liver regeneration (ALR) is one of the key factors contributing to liver growth and regeneration (Gupta and Venugopal, 2018). The specific stimulatory and protective effects of ALR against various injuries have caught the eye of the scientific community, including researchers investigating NAFLD (Nalesnik et al., 2017). Mitochondrial dysfunction is a major contributor to the development of NAFLD, and ALR has critical mitochondrial functions (Kumar et al., 2020). Herein, we focused on the main isoform, 23-kDa ALR, located in mitochondria. This isoform protects hepatocytes against NAFLD *via* the modulation of mitochondrial homeostasis and mitophagy, suppression of oxidative stress, and promotion of cell regeneration.

### Etiology of non-alcoholic fatty liver disease

With economic growth and changing lifestyles, the prevalence of NAFLD has increased rapidly to become a global burden. NAFLD occurs in 25% of the general population, especially in developed countries (Younossi et al., 2016; Cotter and Rinella, 2020; Gallego-Duran et al., 2021). It is estimated that the annual incidence of hepatocellular carcinoma is between 0.5% and 2.6% in patients with NAFLD and cirrhosis (Huang et al., 2021a). Given the vast proportion of patients with NFALD and its complications worldwide, the economic burden is huge.

## Genetic factors in non-alcoholic fatty liver disease

The pathogenesis of NAFLD is not fully understood. Currently, the “multiple- or continuous-hit” hypothesis is the most accepted explanation (Gupta and Venugopal, 2018). An unhealthy lifestyle and its associated metabolic disorders, as well as genetic factors, contribute to the progression of NAFLD. Nonetheless, NAFLD is considered to result from an imbalance of energy metabolism in the liver (Mezhibovsky et al., 2021). Thus far, several genes have been identified to be critically involved in NAFLD.

### Patatin-like phospholipase domain-containing protein 3

Pennacchio et al. and Kim et al. identified the *PNPLA3* gene as the most important genetic factor related to NAFLD to date (Romeo et al., 2008; Moon et al., 2022). The gene variant *PNPLA3*(148M) is a major risk factor for fatty liver, as it promotes steatosis through comparative gene identification 58- (CGI-58-) dependent inhibition of adipose triglyceride lipase (ATGL) during the progression of liver disease. CGI-58 is a cofactor of ATGL that significantly enhances the triglyceride (TG) hydrolase activity. Wang et al. speculated that the accumulation of *PNPLA3*(148M) sequestered the function of CGI-58, thereby limiting its access to ATGL or other lipases (Powell et al., 2019).

### Membrane-bond O-acyltransferase domain-containing 7

A variant of *MBOAT7*, which incorporates arachidonic acid into phosphatidylinositol (PI) (Lee et al., 2012), is also associated with the entire spectrum of NAFLD (Luukkonen et al., 2016; Mancina et al., 2016). The *MBOAT7* gene encodes lysophosphatidylinositol acyltransferase 1 (LPIAT1), which preferentially binds arachidonic acid to PI (Lee et al., 2008), which is a constituent of membrane phospholipids and a precursor of phosphoinositide. Tanaka et al. demonstrated that the depletion of *LPIAT1* in cultured hepatic cells caused a high PI turnover, which continuously produced diacylglycerol, a substrate for TG synthesis. This directly caused TG accumulation and collagen deposition within hepatocytes. Ultimately, this novel lipogenesis pathway is involved in the progression of NAFLD and may be a therapeutic target for NAFLD treatment (Tanaka et al., 2021).

### Transmembrane 6 superfamily antigen 2

The *TM6SF2* gene encodes a protein involved in regulating hepatic TG secretion. A glutamic acid to lysine substitution at amino acid position 167 of the TM6SF2 protein (E167K) disrupts the secretion of very low-density lipoprotein (VLDL). Deletion of *TM6SF2* resulted in abnormal VLDL-TG secretion, which progressed to hepatic steatosis (Carlsson et al., 2020). The lipidation of VLDL is a two-step process, with phospholipids and polyunsaturated fatty acids as key players in the second stage; *TM6SF2* may also be involved in the second step of lipidation (Luo et al., 2022). Luukkonen et al. reported reduced levels of liver polyunsaturated fatty acids, serum TG, and hepatic phosphorylcholine in patients carrying the *TM6SF2*(E167K) variant. Knockdown of *TM6SF2* in Huh7 and HepG2 cell lines reduced the expression of diacylglycerol O-acyltransferase 1 and 2 (Martin et al., 2021), which are two key enzymes in TG synthesis. In conclusion, the function of *TM6SF2* is vital for the lipidation of VLDL (Luukkonen et al., 2017; Luo et al., 2022).

### Lipodystrophy-associated genes

Lipodystrophy syndromes are extremely rare disorders of body fat deficiency associated with potentially serious metabolic complications, including diabetes, hypertriglyceridemia, steatohepatitis, and NAFLD (Brown et al., 2016). Mutations in genes associated with lipodystrophy, such as the peroxisome proliferator-activated receptor-gamma (*PPARγ*), lamin A/C (*LMNA*), and hormone-sensitive lipase genes, are potential therapeutic targets for NAFLD (DiStefano and Gerhard, 2022). PPARγ is part of the nuclear receptor family of transcription factors consisting of PPARγ, PPARα, and PPARδ (Liss and Finck, 2017). PPARγ performs different functions in various cells of the liver. In hepatocytes, PPARγ mediates the expression of adipogenesis genes, such as *AP2* and *CD36*, which induce an increased uptake of free fatty acid (FFA). Simultaneously, the accumulation of FFA promotes intracellular TG accumulation (Chui et al., 2005; Wu et al., 2010). In hepatic macrophages, Kupffer cells (KCs) and monocytes, PPARγ promotes the activation of activated macrophages (M2) while inhibiting the activation of classical macrophages (M1). This reduces the release of inflammatory cytokines, such as tumor necrosis factor-α (TNF-α) and monocyte chemoattractant protein 1, and growth factors such as transforming growth factor-β (TGF-β), leading to reduced inflammation and activation of hepatic stellate cells (HSCs), consequently attenuating fibrosis. PPARγ is also associated with the quiescent phenotype of HSCs, limiting HSC activation and subsequent fibrosis (Skat-Rordam et al., 2019). Mahdi et al. described a 42-year-old female with lipodystrophy and NAFLD due to a pathogenic gene variant *LMNA*(D300N) (Peng et al., 2020). A polymorphism in the promoter of this hormone-sensitive lipase gene was associated with hepatic steatosis, obesity, diabetes, and dyslipidemia. Hsiao et al. found that patients with NAFLD often had complex metabolic abnormalities. Notably, the coexistence of NAFLD and glucose intolerance was shown to have a synergistic effect on increasing the body mass index, serum insulin levels, and homeostatic model assessment of insulin resistance. Body mass index and fat-insulin resistance, but not the homeostatic model assessment of insulin resistance, are consistent indices of insulin resistance in NAFLD studies. Thus, fat-insulin resistance may have the greatest effect on the elevation of serum TG in a state of glucose intolerance (Hsiao et al., 2013).

### Metabolic disorders in non-alcoholic fatty liver disease

With economic growth, the global prevalence of metabolic syndromes, such as obesity, diabetes, and dyslipidemia, increases annually (Iacob and Iacob, 2022). Excessive intake of fructose, refined carbohydrates, sugar-sweetened beverages, saturated fat, and animal protein was identified as a major factor in the development of NAFLD (Parry and Hodson, 2017). For example, regular fructose consumption can induce hepatic lipogenesis and endoplasmic stress, impair fatty acid oxidation, deplete beneficial bacteria in the gut, and cause liver inflammation resulting from the production of uric acid and gut-derived endotoxins (Vos and Lavine, 2013; Jones et al., 2019). The World Health Organization reported that the number of obese people in China was below 0.1 million in 1975 and rose to 43.2 million in 2014, accounting for 16.3% of global obesity. A high-fat and high-carbohydrate diet and unhealthy lifestyle are the main causes of overweight/obesity and impair insulin resistance, which is key to the physiopathology of hepatic steatosis. Moreover, obesity-related hyperlipidemia worsens lipid metabolism disorders and is the most distinct feature of NAFLD. Given the increasing rates of obesity, type 2 diabetes mellitus, and other metabolic syndromes, coupled with an aging population, the incidence of NAFLD is projected to increase dramatically over time (Marjot et al., 2020).

Mitochondrial dysfunction is frequently related to the development of NAFLD. Indeed, structural and functional alterations of mitochondria significantly contribute to changes in cellular lipid metabolism and oxidant stress responses (Auger et al., 2015). Domínguez-Pérez et al. found that cholesterol overload in the mouse liver induced by a high-cholesterol diet led to cholesterol and TG accumulation within hepatocytes, particularly their mitochondria. Moreover, this overload induced remarkable transcriptomic changes, mainly associated with mitochondrial function and dynamics favoring oxidative stress and apoptosis resistance, which could promote transformation (Dominguez-Perez et al., 2019). Dysfunction of hepatocyte endoplasmic reticulum (ER) homeostasis and the disturbance of its interaction with mitochondria also play an important role in NAFLD pathophysiology. The ER uses the unfolded protein response pathway to maintain protein and lipid homeostasis whenever exposed to hyperlipidemia, insulin resistance, inflammation, drugs, or other disturbances (Flessa et al., 2022).

In addition to hepatocytes, KCs and HSCs are associated with the occurrence of NAFLD and progression to NASH during different stages of the NAFLD spectrum (You et al., 2008; Leroux et al., 2012; Teratani et al., 2012). The KCs are tissue-resident cells capable of self-renewal and the maintenance of liver homeostasis. Under normal conditions, KCs tend to suppress inflammation by secreting cytokines such as interleukin 4 (IL-4), IL-10, and IL-13 (Dixon et al., 2013). Hepatocytes express a series of membrane and cytoplasmic pattern recognition receptors, such as Toll-like receptor-4 (TLR-4), all of which stimulate KC activation and trigger a phenotypic switch of macrophages from M2 to M1 (Wan et al., 2014). Activated KCs promote the release of various inflammatory chemokines, including IL-1β, TNF-α, and IL-6 (Musso et al., 2013). These cytokines further recruit large numbers of monocyte-derived infiltrating macrophages to the damaged area, worsening inflammation and hepatocyte injury (Tacke, 2017).

Chronic inflammation sustains these inflammatory stimuli and induces HSCs to initiate fibrotic processes. Located in the space of Disse in the liver, HSCs are physically quiescent cells that function as a store of vitamin A. Following sustained inflammation, HSCs are activated by cytokines and free radicals released from the surrounding cells, such as hepatocytes, T cells, and KCs. Once activated, HSCs undergo a phenotypic switch from adipocyte-like quiescent cells to myogenic cells with increased protein expression of alpha-smooth muscle cell actin (α-SMA) and extracellular matrix (ECM) proteins (Dooley et al., 2000). Increased ECM synthesis and reduced ECM degradation lead to excessive collagen deposition and the progression of fibrosis in the liver.

**FIGURE 1:**
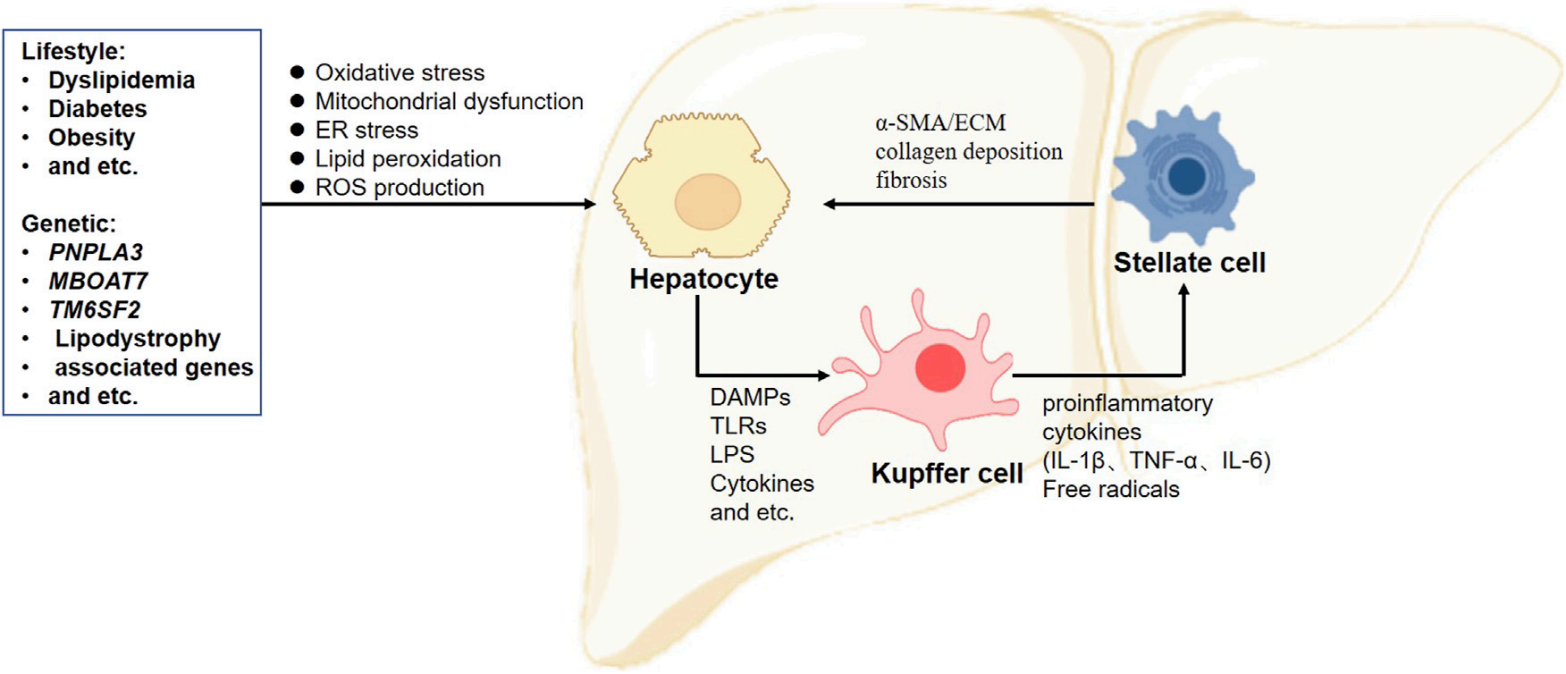
Crosstalk between hepatic cells in the progression of non-alcoholic fatty liver disease. Excessive accumulations of triglyceride, cholesterol, and lipid deposition are considered the first steps to induce hepatocellular lipotoxicity, followed by oxidative stress, peroxidation, mitochondrial dysfunction, and reactive oxygen species (ROS) production. These factors promote hepatocyte death and the release of DAMPs and TLRs, resulting in Kupffer cell activation. Activated Kupffer cells produce a series of proinflammatory cytokines to recruit blood monocytes to strengthen inflammation. When chronic inflammation is sustained, hepatic stellate cells are activated and undergo a phenotype switch from adipocyte-like quiescent cells to myogenic cells with increased expression levels of α-SMA and ECM proteins. Increased ECM synthesis and reduced ECM degradation lead to excessive collagen deposition and fibrosis progression in the liver. α-SMA, alpha-smooth muscle cell actin; DAMPs, damage-associated molecular patterns; ECM, extracellular matrix; ER, endoplasmic reticulum; IL, interleukin; ROS, reactive oxygen species; TNF-α, tumor necrosis factor alpha; TLRs, Toll-like receptors.

Collectively, the crosstalk among hepatic cells substantially contributes to the development of NAFLD (Figure 1). Thus, determining how to protect hepatic cells from death and control the propagation of inflammation is essential for further understanding the pathology of NAFLD.

### Augmenter of liver regeneration

#### Brief introduction

ALR was first identified in 1975 in crude extracts of liver homogenates in weaning rats (LaBrecque and Pesch, 1975). Injection of the purified substance into mice with partial hepatectomy stimulated liver regeneration. Therefore, it was named hepatic regenerative stimulator substance. More recently, it was formally named “augmenter of liver regeneration” (ALR). ALR is widely distributed in the testis, liver, kidney, brain, and other tissues, with maximum expression in the testis and liver (Lisowsky, 1996). Inside the liver, ALR is predominately expressed in hepatocytes and, to a lesser extent, in stellate cells (Gupta and Venugopal, 2018). Regarding subcellular localization, ALR is expressed in the nucleus and cytosol, as well as mitochondria (Weiss et al., 2017). Deletion of ALR was lethal in a yeast system (Becher et al., 1999).

### Gene sequence and protein structure of augmenter of liver regeneration

The human ALR gene (growth factor erv1-like gene, *GFER*) is located on chromosome 16 and consists of three exons and two introns (Hagiya et al., 1994; Lisowsky et al., 1995), comprising a 299-bp 5ʹ untranslated region, a 375-bp coding sequence, and 550-bp 3ʹ untranslated region (Hagiya et al., 1994). A cDNA clone was more than 1.5-kb in length, and *GFER* has a “TATA-less” promoter (Shanks et al., 1993). Thus far, three isoforms of human ALR have been identified.

The human ALR protein yields bands at 15-, 21-, and 23-kDa under reducing conditions, corresponding to 36-, 38-, and 40-kDa under non-reducing conditions, respectively (Dayoub et al., 2011; Dayoub et al., 2013; Gandhi et al., 2015; Weiss et al., 2017; Ibrahim et al., 2018). The 15-kDa ALR is secreted from hepatocytes into the extracellular environment, whereby it displays anti-apoptotic and anti-oxidative properties as well as inflammation- and metabolism-modulating effects (Ibrahim and Weiss, 2019). The 23-kDa ALR is a sulfhydryl oxidase that resides primarily in the mitochondrial intermembrane space (IMS), whereby it exerts liver protection effect against various types of injury (Mordas and Tokatlidis, 2015; Nalesnik et al., 2017; Weng et al., 2017; Jiang et al., 2019). Studies of 21-kDa ALR are limited. In this review, we focused on 23-kDa ALR.

### Regulation of augmenter of liver regeneration gene expression

The promoter region of *GFER* contains sites that bind inducers and repressors that positively or negatively regulate the ALR expression. Inducers include specific protein 1 (SP1), forkhead box A2 (FOXA2), early growth response protein 1 (Egr-1), and hepatocyte nuclear factor 4α (HNF4α). Repressors include activator protein 1/activator protein 4 (AP1/AP4), CCAAT/enhancer binding proteins (C/EBPβ), and HNF4α (Ibrahim et al., 2018). Binding of HNF4α to the promoter region (−209 to −204 bp) reduces *GFER* expression, whereas binding to another site (+421 to +432 bp) induces *GFER* expression (Guo et al., 2008). The downstream inducing effect of HNF4α is diminished upon the activation of the small heterodimer partner protein (SHP) (Ibrahim et al., 2018).

There are two inducing response elements within the *GFER* promoter region. An upstream antioxidant response element (ARE) located between −27 and −19 bp induces *GFER* expression upon binding nuclear factor erythroid 2-related factor 2 (Nrf2) when hepatocytes are exposed to oxidative stress (Dayoub et al., 2013). In addition, a downstream site binding the IL-6 response element binding protein (IL-6-RE-BP) can increase the activating effect of FOXA2 (Dayoub et al., 2010).

**FIGURE 2:**
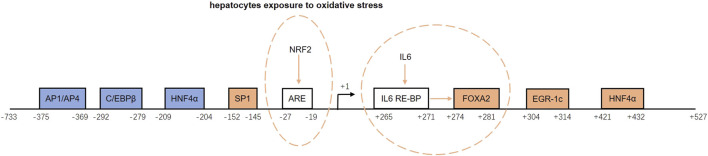
Structure and regulation of the augmenter of liver regeneration (ALR) gene promoter. The blue boxes represent repressors, and the orange boxes are inducers. ARE and IL-6 RE-BP are inducing response elements. NRF2 binds to ARE and induces ALR gene expression when hepatocytes are exposed to oxidative stress. When IL-6 RE-BP is activated by IL-6, it increases the activating effect of FOXA2. AP1/AP4, activator protein 1/activator protein 4; C/EBPβ, CCAAT/enhancer binding protein-beta; HNF4α, hepatocyte nuclear factor 4 alpha; SP1, specific protein 1; ARE, antioxidant response element; NRF2, nuclear factor erythroid 2-related factor 2; IL-6, interleukin-6; IL-6-RE-BP, IL-6 response element binding protein; FOXA2, forkhead box A2; Egr-1, early growth response protein 1.

The promoter structure and regulation of the ALR gene are shown in Figure 2.

### Augmenter of liver regeneration variants

Mutations of *GFER* lead to severe mitochondrial disease. Fonzo et al. identified a c.581G→A homozygous mutation in the *C*-terminus of ALR, which results in a p. R194H substitution in children with autosomal recessive myopathy. At the cellular level, this mutation leads to respiratory chain defects, such as abnormal mitochondrial morphology and unstable mtDNA (Di Fonzo et al., 2009). Daithanker et al. characterized the R194H mutation in the context of enzymological studies of human ALR. The R194H mutation affected the thermal stability of mitochondria, as well their flavin adenine dinucleotide (FAD) binding and sensitivity to protein hydrolysis (Daithankar et al., 2010). The yeast ortholog Erv1p, a key protein in the mitochondrial disulfide relay system, oxidizes the disulfide carrier mitochondrial import and assembly protein 40 (Mia40), which in turn transfers disulfide bonds to newly synthesized small cysteine proteins in the IMS. Erv1p is then re-oxidized to transfer its electrons to molecular oxygen through interactions with cytochrome C and cytochrome C oxidase, linking the disulfide relay system to respiratory chain activity. Erv1p depletion prevents the import of these essential proteins, leading to mtDNA aberrations and abnormal mitochondrial morphology. Rat and human ALR proteins act as sulfhydryl oxidase and may play a role similar to that of yeast Erv1p (Di Fonzo et al., 2009). The features of ALR variants are summarized in Table 1.

**TABLE 1:** Summary of human ALR variants.

Allelic variants	Type of mutations	Clinical features	References
*GFER*, ARG194HIS	arg-to-his(R194H) substitution	Myopathy, mitochondrial progressive, with congenital cataract and developmental delay	Di Fonzo et al. (2009)
*GFER*, GLN125TER	gln-to-ter(Q125X) substitution	Myopathy, mitochondrial progressive, with congenital cataract and developmental delay	Calderwood et al. (2016)
*GFER*, 1-BP DEL, 219C	Frameshift and premature termination codon	Myopathy, mitochondrial progressive, with congenital cataract and developmental delay	Nambot et al. (2017)
*GFER*		Myopathy, mitochondrial progressive, with congenital cataract and developmental delay	
*GFER*, 1-BP DEL, 217G		Myopathy, mitochondrial progressive, with congenital cataract and developmental delay	

ALR, augmenter of liver regeneration; ARG, arginine; BP, base pair; DEL, deletion; *GFER*, growth factor erv1-like gene; GLN, glutamine; HIS, histidine; TER, threonine.

### Protective role of augmenter of liver regeneration in hepatocytes in non-alcoholic fatty liver disease

At the organelle level, 23-kDa ALR is mainly located in the IMS, whereby it regulates mitochondrial biogenesis and function. The FAD-dependent sulfhydryl oxidase activity of ALR allows it to enhance the oxidative phosphorylation capacity of mitochondria (Daithankar et al., 2009). Compelling evidence indicates that ALR inhibits apoptosis, promotes hepatocyte regeneration, and prohibits fibrotic progression in various murine models of NAFLD (Xiao et al., 2015; Xu et al., 2016; Weiss et al., 2017; Kumar et al., 2020; Wang et al., 2020). All these beneficial effects of ALR in hepatocytes are directly or indirectly related to its regulation of mitochondria. Below, we focus on the protective effects of ALR in hepatocytes in the aspects of cell death, regeneration, and anti-fibrosis in NAFLD.

### Augmenter of liver regeneration in hepatocyte apoptosis and autophagy

Excessive accumulation of TG, cholesterol, and lipid deposition are considered the first steps to induce hepatocellular lipotoxicity, followed by oxidative stress, lipid peroxidation, mitochondrial dysfunction, and excessive reactive oxygen species production (Dewidar et al., 2020). Taking cholesterol accumulation as an example, the activation of the adenosine monophosphate-activated protein kinase (AMPK) signaling pathway improves insulin resistance and lipid accumulation (Hsiao et al., 2013). Wang et al. showed that cholesterol accumulation within hepatocytes can be regulated by ALR *via* the liver kinase B1- (LKB1-) AMPK-sterol regulatory element binding protein 2- (SREBP2-) low-density lipoprotein receptor pathway. LKB1 is an upstream activator of AMPK. Knockdown of ALR expression inhibits LKB1 phosphorylation, leading to AMPK inactivation and SREBP2 maturation/nuclear translocation. SREBP2 and low-density lipoprotein receptor actions are closely associated with cholesterol accumulation within hepatocytes. Thus, alterations in these events can lead to extensive cholesterol accumulation and the development of lipid metabolism disorders (Wang et al., 2020).

Results from many research groups have illustrated that ALR inhibits apoptosis and helps overcome cell injury induced by CCl_4_, ethanol, and other toxic factors. Studies in liver cells show that the downregulation of ALR results in increased activation of caspase-3 and caspase-9, an increased ratio of Bax/Bcl-2 expression, and reduced ATP content (Francavilla et al., 2014; Zhang et al., 2014; Dong et al., 2021). Beyond its role in cell apoptosis, there has been considerable evidence indicating a role for ALR in reducing autophagy. For example, in an *in vivo* ethanol-induced acute liver injury mouse model, the downregulation of ALR attenuated hepatotoxicity by activating autophagy, and *in vitro* experiments in the HepG2 cell line showed that protection was mediated by the inactivation of the Akt/mTOR pathway (Liu et al., 2019).

### Augmenter of liver regeneration in mitochondrial homeostasis

From mechanistic insights, mitochondria play a central role in hepatocyte survival. The transfection of ALR into steatotic hepatocytes upregulates carnitine palmitoyl transferase 1 (CPT1) expression to enhance long-chain fatty acid transport into mitochondria for usage (Xiao et al., 2015). Dynamin-related protein 1 (Drp1) is one of the major pro-fission proteins to clear damaged mitochondrial debris and govern mitochondrial homeostasis (Jin et al., 2021). In a murine model of hepatic ischemic reperfusion injury, the Drp1 activity increased, which promoted mitochondrial fission. Binding with transcription factor Yin Yang-1 (YY1) with ALR prohibited YY1 nuclear translocation and transcriptional activation. As one of the target genes of YY1, UBA2 is a subunit of the SUMO-E1 enzyme and catalyzes Drp1 SUMOylation. By doing so, ALR attenuated mitochondrial fission and retained its function (Huang et al., 2021b). Mitofusin-2 (Mfn-2) is an essential GTPase-related mitochondrial dynamics protein. In the same murine model of hepatic ischemic reperfusion injury, ALR administration accelerated Parkin translocation for transcriptional activation of Mfn2, leading to enhanced mitophagy (Kong et al., 2022).

Most soluble IMS proteins rely on a mitochondria-targeting sequence for import, and ALR participates in protein import and export by cooperating with Mia40 (Finger and Riemer, 2020). Mia40 is reduced during the process of disulfide bond formation, and ALR can re-oxidize reduced Mia40 to make it available for the next round of disulfide bond formation (Grumbt et al., 2007; Bien et al., 2010; Banci et al., 2011). This function of ALR ensures adequate protein folding during import and export to the IMS, which is necessary for functioning mitochondria. Recent progress in the field revealed that the coiled-coil-helix-coiled-coil-helix domain-containing 4 (CHCHD4) proteins, the evolutionarily conserved human homolog of yeast Mia40, control antioxidant responses and lipid homeostasis (Reinhardt et al., 2020). Hence, CHCHD4/Mia40 could be a novel target for NAFLD investigations.

Gandhi et al. successfully developed mice with liver-specific depletion of ALR (ALR-L-KO), which showed that a lack of ALR accelerated the development of steatohepatitis and hepatocellular carcinoma (Gandhi et al., 2015). Two weeks after birth, the ALR-L-KO mice showed reduced mitochondrial respiratory function, increased oxidative stress, and extensive steatosis and apoptosis. Furthermore, ALR depletion resulted in decreased expression of genes involved in lipid metabolism, such as CPT1α, and ATP synthesis, such as ATP synthase subunit ATP5G1. This model provides a useful tool to investigate the pathogenesis of steatohepatitis and its complications and further showed that ALR is required for mitochondrial function and lipid homeostasis in the liver.

### The anti-oxidative properties of augmenter of liver regeneration

Peroxisome proliferator-activated receptor-alpha (PPAR-α), CPT1-α, peroxisomal membrane protein 70 (PMP70), and acyl-CoA oxidase 1 (ACOX1) are a series of antioxidant proteins, which are targeted by miR540 (Kumar et al., 2019). In ALR-deficient hepatocytes, the miR540 expression was increased and the expression levels of PPARα, PMP70, ACOX1, and CPT1α were decreased. In contrast, antioxidant *N*-acetylcysteine and recombinant ALR rescued anti-oxidative stress responses by suppressing miR-540 expression and lipid accumulation in ALR-deficient hepatocytes. In agreement with these results, the exogenous administration of recombinant ALR to ALR^−/−^KO mice inhibited miR-540 expression and steatosis (Kumar et al., 2019).

### Augmenter of liver regeneration in endoplasmic reticulum stress

FFA can induce steatosis and lipotoxicity, which are correlated with the severity of NAFLD. Moreover, the involvement of ER stress in lipotoxicity has been reported (Malhi and Kaufman, 2011). Xu et al. investigated the role of endogenous and exogenous ALR for FFA-induced ER stress and lipotoxicity. When hepatocytes treated with ALR or expressing ALR were incubated with palmitic acid *in vitro*, caspase-3 activity and Bax protein expression were reduced, therefore reducing lipotoxicity. These results indicate that ALR exerted its lipid-lowering and anti-apoptotic actions by elevating the mitochondrial FFA transporter CPT1α, increasing toxic FFA β-oxidation in mitochondria, and decreasing the delivery of toxic FFA metabolites. *In vivo*, reduced mRNA levels of ALR and FOXA2 (a transcription factor inducing ALR expression) were found in mice fed a high-fat diet, human patients with steatosis, and NASH liver samples. These results demonstrate the role of ALR in reducing lipid deposition and increasing β-oxidation in patients with NASH (Xu et al., 2016). Xiao et al. further confirmed that the protective role of ALR against steatosis occurred *via* the inhibition of calcium transport from the ER to mitochondria, and the inhibition of ER stress by ALR was associated with an interrupted interaction between Bcl2 and the inositol 1,4,5-trisphosphate receptor (IP3R) (Xiao et al., 2018).

### Augmenter of liver regeneration in hepatocyte regeneration

The removal of 75% of rat liver tissue led to increased ALR mRNA expression levels in hepatocytes after 12 h, but DNA synthesis in liver tissue reached a peak 24 h later (Francavilla et al., 2014). This suggests that ALR is a significant factor in the process of liver regeneration (Fausto, 1991; Gandhi et al., 1999; Polimeno et al., 2011). The use of MitoBloCK-6 to pharmacologically inhibit ALR reduced the proliferation of hepatocellular carcinoma cells, an effect that links ALR function to mitochondrial iron homeostasis (Kabiri et al., 2021). Silencing of ALR inhibited the proliferation and triggered the apoptosis of U266 human multiple myeloma cells (Zeng et al., 2017). Conversely, ALR overexpression in hepatic cells enhanced cell proliferation *via* the microRNA-26a/p-Akt/cyclin D1 pathway (Gupta et al., 2019a).

Kupffer cells (KCs) play a protective role in liver regeneration (Selzner et al., 2003), and a relationship between KCs and ALR has been reported. Yang et al. suggested that the activation of KCs was another mechanism by which ALR stimulates hepatocyte proliferation because there are high-affinity receptors for ALR on hepatic KCs, and ALR can stimulate KC proliferation (Wang et al., 2006). Similarly, when hepatocytes were co-cultured with KCs, the levels of hepatocyte DNA and protein in the supernatant were significantly increased (Kinoshita et al., 2005). These events indicate that ALR can regulate KCs to secrete certain growth factors which promote hepatocyte proliferation.

Acute response cytokines, such as IL-6 and TNF-α, are mainly released from KCs and are associated with hepatocyte proliferation (Olthoff, 2002). As described above, ALR binds to KCs *via* high-affinity receptors. The activation of KCs induces the release of various cytokines that trigger hepatocyte proliferation.

### Anti-fibrotic property of augmenter of liver regeneration

Therefore, there is no evidence for a direct anti-fibrotic effect of ALR on hepatocytes. Nevertheless, ALR inhibits fibrotic progression in the liver by suppressing hepatic stellate cell (HSC) activation in NAFLD.

Among all inflammatory cytokines, TGF-β1 is the most potent stimulator of HSC activation (Mu et al., 2018; Xiang et al., 2020; Cheng et al., 2021). The binding of TGF-β1 to its receptors on HSCs results in the phosphorylation of several serine and threonine residues, which stimulate Smad2 and Smad3 kinase activation and the formation of the Smad2/Smad3/Smad4 complex. This complex is then translocated into the nucleus, whereby it transcriptionally activates the expression of fibrotic genes, including the mitogen-activated protein kinase 1 (MAPK), phosphoinositide 3-kinase (PI3K), nuclear factor-κB (NF-κB), NADPH oxidase, and connective tissue growth factor genes (Pei and Li, 2021). In LX-12 cells treated with TGF-β1, miR-181 was upregulated, further increasing TGF-β receptor II expression on HSCs to potentiate fibrotic pathways. However, when LX-12 cells were transfected with an ALR plasmid, the overexpression of ALR counteracted TGF-β-induced miR-181 and TGF-β receptor II expression (Gupta et al., 2019b). Likewise, in cultivated renal tubular cells, the addition of human recombinant ALR decreased the TGF-β receptor II expression and phosphorylation of Smad2 and NF-κB (Liao et al., 2014).

Metalloproteinases, which play a dominant role in ECM degradation, are inhibited by tissue inhibitors of metalloproteinase (TIMP). Of the four TIMPs, only TIMP-1 and TIMP-2 are detected in liver tissue, with TIMP-1 expression being more pronounced than TIMP-2. In a rat hepatic fibrosis model induced by porcine serum injection, the administration of ALR plasmid decreased the expression levels of TIMP-1 mRNA and protein and was accompanied by reduced deposition of collagen I and collagen II in the liver (Li et al., 2005).

Cell motility is ATP-consuming and mediated by microfilament assembly. *In vitro*, ALR knockdown by shRNA promoted mitochondrial fission and elongation, which led to enhanced ATP production for HSC migration. Moreover, the proportions of F-actin and G-actin were higher in ALR-deficient HSCs following shRNA transfection. Conversely, ALR overexpression slowed HSC migration by reducing energy supply and inhibiting mitochondrial fusion (Ai et al., 2018).

**FIGURE 3:**
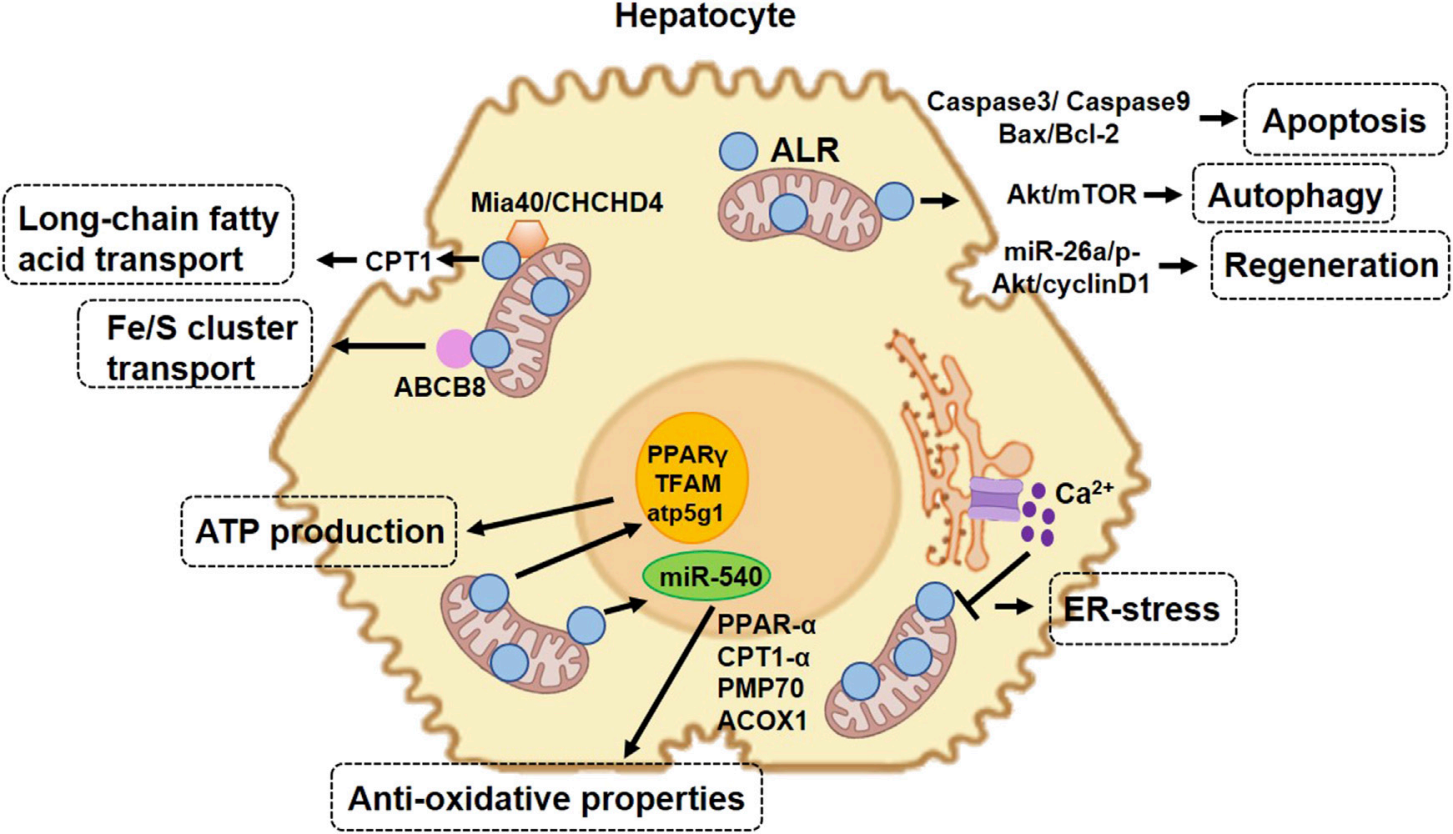
Protective role of augmenter of liver regeneration (ALR) in hepatocytes in non-alcoholic fatty liver disease. 23-kDa ALR is located in the intermembrane space of mitochondria and regulates hepatocyte function through different mechanisms. In the aspect of mitochondrial homeostasis, ALR upregulates the expression of CPT1, increasing the transport of long-chain fatty acids into mitochondria. In the aspect of anti-oxidative stress, ALR cooperates with Mia40 ensuring the adequate folding of IMS-proteins during import and export to IMS and functioning in mitochondria. ALR binds to ABCB8 and plays a role in Fe/S cluster transport. In addition, ALR suppresses the expression of miR-540, which increases the expression of several proteins involved in anti-oxidative stress. ALR also promotes cell proliferation and liver regeneration and maintains stem cell “stemness” and survival. Overexpression of ALR decreases the expression of caspase-3, thereby decreasing cell apoptosis. CPT1, carnitine palmitoyl transferase-1; IMS, mitochondrial intermembrane space; Mia40, mitochondrial import and assembly protein 40; CHCHD4, coiled-coil-helix-coiled-coil-helix domain-containing 4; PPAR-α, peroxisome proliferator-activated receptor alpha; PMP70, peroxisomal membrane protein 70; ACOX1, acyl-CoA oxidase 1; Fe/S, iron/sulfur clusters; ABCB8, ATP-binding cassette B8; miR-540, microRNA 540; TFAM, mitochondrial transcription factor A.

The proposed mechanism by which ALR protects hepatocytes in NAFLD is summarized in Figure 3.

### Perspective

Nearly 50 years of research on ALR has consistently demonstrated its involvement in the spectrum of NAFLD. Despite this progress, how ALR expression is regulated in the context of NAFLD is not well defined. Moreover, most ALR studies focus on the liver. Thus, whether ALR in other organs, such as the kidney and brain, communicate with the liver to participate in NAFLD remains an open question. Additionally, although 21-kDa ALR is one of the main isoforms, its role is not fully understood. There are still mysteries surrounding ALR worth exploring in the future.

In conclusion, ALR promotes mitochondrial homeostasis, protects hepatocyte survival and function, and suppresses macrophage and HSC activation. Collectively, these features make ALR a potential therapeutic target for the treatment of NAFLD.
